# Predictors of Graft Failure in Young Active Patients Undergoing Hamstring Autograft Anterior Cruciate Ligament Reconstruction With or Without a Lateral Extra-articular Tenodesis: The Stability Experience

**DOI:** 10.1177/03635465211061150

**Published:** 2022-01-20

**Authors:** Andrew D. Firth, Dianne M. Bryant, Robert Litchfield, Robert G. McCormack, Mark Heard, Peter B. MacDonald, Tim Spalding, Peter C.M. Verdonk, Devin Peterson, Davide Bardana, Alex Rezansoff, STABILITY Study Group, Alan M.J. Getgood, Kevin Willits, Trevor Birmingham, Chris Hewison, Stacey Wanlin, Ryan Pinto, Ashley Martindale, Lindsey O’Neill, Morgan Jennings, Michal Daniluk, Dory Boyer, Mauri Zomar, Karyn Moon, Raely Moon, Brenda Fan, Bindu Mohan, Gregory M. Buchko, Laurie A. Hiemstra, Sarah Kerslake, Jeremy Tynedal, Greg Stranges, Sheila Mcrae, LeeAnne Gullett, Holly Brown, Alexandra Legary, Alison Longo, Mat Christian, Celeste Ferguson, Nick Mohtadi, Rhamona Barber, Denise Chan, Caitlin Campbell, Alexandra Garven, Karen Pulsifer, Michelle Mayer, Nicole Simunovic, Andrew Duong, David Robinson, David Levy, Matt Skelly, Ajaykumar Shanmugaraj, Fiona Howells, Murray Tough, Pete Thompson, Andrew Metcalfe, Laura Asplin, Alisen Dube, Louise Clarkson, Jaclyn Brown, Alison Bolsover, Carolyn Bradshaw, Larissa Belgrove, Francis Milan, Sylvia Turner, Sarah Verdugo, Janet Lowe, Debra Dunne, Kerri McGowan, Charlie-Marie Suddens, Geert Declerq, Kristien Vuylsteke, Mieke Van Haver

**Affiliations:** London Health Sciences Centre, Western University, Fowler Kennedy Sport Medicine Clinic, London, Canada; Fraser Orthopaedic Institute, New Westminster, Canada; Banff Sport Medicine, Banff, Canada; Pan Am Clinic, Winnipeg, Canada; University Hospitals Coventry Warwickshire NHS Trust, Coventry, UK; Antwerp Orthopaedic Center, Ghent, Belgium; McMaster University, Hamilton, Canada; Queens University, Kingston, Canada; Sport Medicine Centre, University of Calgary, Calgary, Canada; London Health Sciences Centre, Western University, Fowler Kennedy Sport Medicine Clinic, London, Canada; Fraser Orthopaedic Institute, New Westminster, Canada; Banff Sport Medicine, Banff, Canada; Pan Am Clinic, Winnipeg, Canada; Sport Medicine Centre, University of Calgary, Calgary, Canada; McMaster University, Hamilton, Canada; Queens University, Kingston, Canada; University Hospitals Coventry Warwickshire NHS Trust, Coventry, UK; Antwerp Orthopaedic Center, Ghent, Belgium; Investigation performed at the Fowler Kennedy Sport Medicine Clinic, Western University, London, Ontario, Canada

**Keywords:** anterior cruciate ligament reconstruction, lateral extra-articular tenodesis, predictors, ACLR failure

## Abstract

**Background:**

Anterior cruciate ligament (ACL) reconstruction (ACLR) has higher failure rates in young active patients returning to sports as compared with older, less active individuals. Augmentation of ACLR with an anterolateral procedure has been shown to reduce failure rates; however, indications for this procedure have yet to be clearly defined.

**Purpose/Hypothesis:**

The purpose of this study was to identify predictors of ACL graft failure in high-risk patients and determine key indications for when hamstring ACLR should be augmented by a lateral extra-articular tenodesis (LET). We hypothesized that different preoperative characteristics and surgical variables may be associated with graft failure characterized by asymmetric pivot shift and graft rupture.

**Study Design:**

Case-control study; Level of evidence, 3.

**Methods:**

Data were obtained from the Stability 1 Study, a multicenter randomized controlled trial of young active patients undergoing autologous hamstring ACLR with or without a LET. We performed 2 multivariable logistic regression analyses, with asymmetric pivot shift and graft rupture as the dependent variables. The following were included as predictors: LET, age, sex, graft diameter, tear chronicity, preoperative high-grade knee laxity, preoperative hyperextension on the contralateral side, medial meniscal repair/excision, lateral meniscal repair/excision, posterior tibial slope angle, and return-to-sports exposure time and level.

**Results:**

Of the 618 patients in the Stability 1 Study, 568 with a mean age of 18.8 years (292 female; 51.4%) were included in this analysis. Asymmetric pivot shift occurred in 152 (26.8%) and graft rupture in 43 (7.6%). The addition of a LET (odds ratio [OR], 0.56; 95% CI, 0.37-0.83) and increased graft diameter (OR, 0.62; 95% CI, 0.44-0.87) were significantly associated with lower odds of asymmetric pivot shift. The addition of a LET (OR, 0.40; 95% CI, 0.18-0.91) and older age (OR, 0.83; 95% CI, 0.72-0.96) significantly reduced the odds of graft rupture, while greater tibial slope (OR, 1.15; 95% CI, 1.01-1.32), preoperative high-grade knee laxity (OR, 3.27; 95% CI, 1.45-7.41), and greater exposure time to sport (ie, earlier return to sport) (OR, 1.18; 95% CI, 1.08-1.29) were significantly associated with greater odds of rupture.

**Conclusion:**

The addition of a LET and larger graft diameter were significantly associated with reduced odds of asymmetric pivot shift. Adding a LET was protective of graft rupture, while younger age, greater posterior tibial slope, high-grade knee laxity, and earlier return to sport were associated with increased odds of graft rupture. Orthopaedic surgeons should consider supplementing hamstring autograft ACLR with a LET in young active patients with morphological characteristics that make them at high risk of reinjury.

There are many studies detailing the successful outcome of anterior cruciate ligament (ACL) reconstruction (ACLR); however, there are also many reports of unsatisfactorily high rates of failure, particularly in the younger athlete.^1,24,27,53,59^ In a systematic review, Wiggins et al^61^ showed that athletes aged <25 years who return to sport (RTS) after ACLR have a reported ipsilateral failure rate between 7% and 14%. Age has been a significant predictor of ACLR failure in multiple studies.^25,28,49,53^ The Multicenter Orthopaedic Outcomes Network (MOON) knee group showed that the odds of ACLR failure decrease by 9% for every year increase in age.^23^ Webster et al^59^ also showed that patients <20 years old had a 30% cumulative risk of ACL reinjury or contralateral knee ACL injury in the first 2 years after reconstruction. Other risk factors for ACLR failure have included increased posterior tibial slope,^58^ meniscal deficiency,^42^ graft size,^28^ and graft choice.^31^

Anterolateral-based procedures, such as lateral extra-articular tenodesis (LET) or the newer anterolateral ligament reconstruction techniques, have emerged as surgical methods to attempt to reduce persistent anterolateral rotatory laxity and ACL graft failure, particularly in patients who may be at high risk of graft failure. A number of theories have postulated why an anterolateral procedure can reduce graft failure. The reduction of persistent rotatory laxity is the most obvious. However, as shown by Engebretsen et al^10^ and more recently Marom et al,^32^ the addition of a LET results in reduced graft forces after ACLR. This may provide some protection during the graft healing and maturation phases. Unfortunately, there is a paucity of literature regarding the indications for adding an anterolateral procedure during ACLR. Two recent consensus articles outlined potential indications for the augmentation of ACLR with an anterolateral procedure.^11,50^ The first included revision ACLR, increased posterior tibial slope, generalized ligamentous laxity (or knee hyperextension >10°), young age, and return to contact pivoting sports.^50^ The second targeted revision ACLR, high-grade pivot shift, Segond fracture, participation in pivoting sports, and hyperlaxity as primary criteria and contralateral ACL rupture, Lachman test displacement >7 mm, deep lateral femoral notch sign, and age <25 years as secondary criteria.^11^ However, both studies were based on level 5 evidence and would suggest that nearly all patients undergoing ACLR should have an anterolateral procedure augmentation. Consequently, given the lower level of evidence behind these position statements and therefore the potentially erroneous conclusions that have been made regarding the addition of anterolateral procedures, studies with higher evidence levels are necessary to determine the appropriate indications for lateral augmentation during ACLR. It is clear that more robust evidence is required to guide surgeons and patients to decide when an anterolateral augmentation of ACLR is required.

We recently performed a multicenter randomized clinical trial in which patients aged ≤25 years who were deemed as being at high risk of reinjury were treated with hamstring tendon autograft ACLR with or without a LET (Stability 1 Study).^12^ At 2 years after surgery, the addition of the LET resulted in a 60% relative risk reduction of graft failure as compared with the ACLR alone.^14^ The purpose of the present study was to (1) identify preoperative variables associated with persistent rotatory laxity or graft rupture in high-risk patients from the Stability 1 Study and (2) determine key indications to inform surgeons when hamstring autograft ACLR should be supplemented with a LET. The hypothesis of this study was that different patient characteristics and surgical variables may be associated with graft failure characterized by asymmetric pivot shift or graft rupture.

## Methods

Data for this analysis were obtained from the Stability 1 Study (ClinicalTrials.gov: NCT02018354). An overall 618 patients between the ages of 15 and 25 years were recruited from 9 centers (7 in Canada, 2 in Europe) and then followed for 2 years postoperatively. Patients underwent clinical assessment and completed patient-reported outcome measures at postoperative 3, 6, 12, and 24 months. The study was approved by the Western University Research Ethics Board and the local board at each institution. A detailed study protocol and results have been published.^12-14^

### Outcomes

The primary outcome in the Stability 1 Study was clinical failure of the ACLR, defined as (1) persistent grade 1 pivot shift at multiple visits, (2) grade ≥2 pivot shift at any visit, or (3) graft rupture confirmed arthroscopically or on magnetic resonance imaging.^2^ The pivot-shift test, which has been shown to be specific (97%-99%; sensitivity, 14%-48%) for detection of ACL rupture,^46^ was completed at each visit by a trained, blinded member of the surgical team and graded according to the International Knee Documentation Committee (IKDC) form.^18^ A positive pivot-shift test finding correlates with worse functional outcome^3,26^ and is widely accepted as a marker of failed ACLR.^33-35^

### Predictors

We selected 12 predictors for our analyses based on previous findings and clinical hypotheses: age,^25,28,49^ sex,^31^ treatment group (ACLR alone or ACLR + LET),^14^ preoperative knee hyperextension on the contralateral side,^8^ graft diameter,^28^ posterior tibial slope,^58^ time from injury to surgery (months),^48^ preoperative high-grade knee laxity,^29,30^ and meniscal treatment status (medial repair, medial excision, lateral repair, lateral excision).^42^

Patients reported age, sex, and date of injury on a preoperative questionnaire. Age was included as a continuous variable to assess the effect of a 1-year increase in age. Tear chronicity was calculated as the interval in months between the date of injury and date of surgery. Passive hyperextension >10° was measured on both knees preoperatively as part of the Beighton score. Because the degree of hyperextension between uninjured knees is highly correlated and the surgical knee in some patients was locked or otherwise unable to demonstrate preinjury extension, we used the measurement from the nonoperative knee. Pivot-shift and Lachman tests were performed with the patient under anesthesia by the operating surgeon at the time of surgery according to the IKDC guidelines.^18^ The presence of a grade 3 Lachman sign (>10-mm difference vs the other side) or grade 3 pivot-shift result (+++ gross) was used to define high-grade knee laxity, similar to previous research by the MOON group.^29,30^ The posterior tibial slope for all patients was measured on a true lateral radiograph taken preoperatively by 1 fellowship-trained orthopaedic surgeon according to the technique used by Webb et al.^58^ Tibial slope was included as a continuous variable to assess the effect of a 1° increase in tibial slope.

Patients were randomized intraoperatively to ACLR alone or ACLR + LET. The type of procedure performed, the ACL graft diameter, and the presence and treatment of meniscal tears were documented on the standardized surgical report forms by the operating surgeon.

### Confounding Variables

Patients reported their primary sports and participation levels (none, recreational, competitive, varsity, elite) preoperatively. Postoperatively, patients were given an RTS questionnaire and asked to indicate when they returned to sports and whether at a higher, similar, or lower level. We classified those who returned to competitive, varsity, or elite sports as high level; those returning to recreational sports as low level; and those who did not return at all. Patients were also classified by their primary sports as high or low risk. High-risk sports were defined as those that required cutting, pivoting, or landing from jumps (eg, soccer, basketball, volleyball) while low-risk sports did not (eg, swimming, running). Exposure time—the number of months that patients were playing sports during the study period—was determined by subtracting the postoperative RTS month from the total follow-up period of 24 months. For example, a patient who returned to sports 6 months postoperatively would have an exposure time of 18 months, and a patient who did not RTS would have an exposure time of zero months. Exposure time was included as a continuous predictor to assess the effect of a 1-month increase in exposure time. Early return to knee-strenuous sports and higher activity level have been shown to be related to increased risk of graft failure.^2,5,23^

### Statistical Analysis

The primary outcome variable was separated into 2 groups by distinct event: asymmetric pivot shift (ie, a grade 1+ pivot shift at multiple visits or a grade 2+ pivot shift at any visit that did not meet the definition of graft rupture) and graft rupture. Different factors may be associated with each event. We performed multivariable logistic regression to predict asymmetric pivot shift and graft rupture using the all-enter method with 11 of 12 predictors. Radiographs were not available for 55 patients; therefore, tibial slope was added in a second step to show the effect of removing these patients and adding slope to the analysis. The graft rupture model was adjusted for the number of postoperative months in which patients returned to sport and the level at which they returned. Potential interactions were evaluated between treatment group and other predictors to determine whether they influenced the effectiveness of adding a LET.

To check the assumptions for logistic regression, locally weighted scatterplot smoothing curves were used to assess linearity between continuous predictors and the log odds. The variance inflation factor was used to detect multicollinearity between predictors. A variance inflation factor >2 required investigation, and a variance inflation factor >10 required the removal of predictors causing multicollinearity.^17^ Outliers and influential points were identified using DFbeta values >0.10,^6^ with planned sensitivity analyses to determine whether removing influential points changed the contribution of each predictor.

We pared the models down to include only important predictors, removing variables where *P* > .30 and presenting odds ratios (ORs) and 95% CIs for each model. The Hosmer-Lemeshow goodness-of-fit test and area under the curve (AUC) for a receiver operating characteristic (ROC) curve were used to assess model fit and its ability to correctly classify individuals. To inform our second objective to determine patients for whom the addition of a LET may be most beneficial, predictors that could not be measured pre- or intraoperatively were removed (ie, RTS level and time). The relationship between these predictors and the addition of a LET were explored by calculating the risk ratio of graft rupture for different thresholds of each variable by treatment group. Last, the predicted probabilities of graft rupture for patients from the Stability 1 Study were determined and presented in graphical form for ease of interpretation. All statistical analyses were performed using Stata 15.1 (StataCorp).^55^

## Results

A total of 618 patients were recruited for the Stability 1 Study, and 587 patients (95.0%) had outcome data available at postoperative 2 years. Nineteen (3.2%) of the remaining patients were missing predictor variables and were removed from this analysis. The characteristics of the 568 patients that we included are presented in Table 1.

**Table 1:** Characteristics of Stability 1 Patients Included in This Analysis^*a*^

Characteristic	Stability 1 Cohort (N = 568)
LET group	282 (49.7)
Age, y	18.8 ± 3.2
Female	292 (51.4)
Knee hyperextension	192 (33.8)
Tear chronicity, mo^*b*^	5 [5.7]
Graft diameter, mm	8.1 ± 0.6
Medial meniscal	
Repair	188 (33.1)
Excision	55 (9.7)
Lateral meniscal	
Repair	91 (16.0)
Excision	130 (22.9)
Posterior tibial slope, deg	9.0 ± 2.7
Preoperative high-grade knee laxity	120 (21.1)
Exposure time, mo	11.2 ± 6.0
RTS level	
None	74 (13.0)
Low risk	98 (17.3)
High risk, low level	152 (26.8)
High risk, high level	244 (43.0)

a Values are presented as No. (%) or mean ± SD. LET, lateral extra-articular tenodesis; RTS, return to sport.

b Median [interquartile range].

Of the available patients, 152 (26.8%) had asymmetric pivot shift and 43 (7.6%) had graft rupture. Adding tibial slope to the 11 predictors in the asymmetric pivot-shift model removed 55 patients from the analysis owing to missing data; the effect of this decision on predictor estimates and precision in the full model is provided in Table 2.

**Table 2:** Odds Ratios (SE) for the Asymmetric Pivot-Shift Model Before and After Including Tibial Slope^*a*^

	Excluding Tibial Slope (N = 568)		Including Tibial Slope (n = 513)	
Predictor Variable	Odds Ratio	SE	Odds Ratio	SE
ACL + LET	0.57	0.11	0.57	0.12
Age	0.95	0.03	0.95	0.03
Female sex	0.99	0.23	1.00	0.24
Knee hyperextension	1.24	0.25	1.38	0.30
Graft diameter	0.60	0.11	0.62	0.12
Medial meniscal				
Repair	1.17	0.25	1.33	0.30
Excision	1.75	0.54	1.58	0.55
Lateral meniscal				
Repair	1.27	0.32	1.34	0.34
Excision	1.25	0.28	1.18	0.29
High-grade knee laxity	0.87	0.21	0.85	0.22
Tibial slope	—	—	1.08	0.04

a ACL, anterior cruciate ligament; LET, lateral extra-articular tenodesis.

The model met all assumptions, and 3 outliers were identified; however, removing them did not change the model estimates or statistical significance. All variance inflation factor values were <2, indicating no multicollinearity among predictors, and no significant interaction terms were identified between treatment and other predictors. Table 3 presents the pared-down model with ORs and 95% CIs for important predictors that remained.

**Table 3:** Predictors of Asymmetric Pivot Shift After Paring Down the Model Using *P <* .30^*a*^

Predictor Variable	Odds Ratio	95% CI	*P* Value^*b*^
ACL + LET	0.56	0.37-0.83	**.004**
Age	0.95	0.89-1.02	.14
Knee hyperextension	1.39	0.91-2.10	.13
Graft diameter	0.62	0.44-0.87	**.005**
Medial meniscal			
Repair	1.30	0.85-1.99	.23
Excision	1.55	0.79-3.06	.19
Tibial slope	1.07	1.00-1.15	.06

a Effect of age per 1-year increase. Effect of graft diameter per 1-mm increase. Effect of tibial slope per 1° increase. ACL + LET, knee hyperextension, and medial meniscal repair and excision included as dichotomous variables (yes/no). ACL, anterior cruciate ligament; LET, lateral extra-articular tenodesis.

b Bold indicates statistical significance.

Adding a LET (OR, 0.56; 95% CI, 0.37-0.83) and increasing graft diameter (OR, 0.62; 95% CI, 0.44-0.87) significantly decrease the odds of an asymmetric pivot shift.

Increasing age remained in the model and was associated with decreased odds of positive pivot-shift result, although it was not statistically significant (*P* > .05). Knee hyperextension, medial meniscal repair, medial meniscal excision, and greater tibial slope were associated with increased odds of asymmetric pivot, although none of these variables were statistically significant (*P* > .05). The Hosmer-Lemeshow test returned a nonsignificant result (*P* = .52), indicating adequate model fit, and the AUC for the ROC curve was 0.64.

The model for graft rupture was performed, including the 12 factors adjusted for RTS time and level. Sport risk and level were combined into 5 categories: (1) no RTS; (2) low risk, low level; (3) low risk, high level; (4) high risk, low level; and (5) high risk, high level. Very few patients returned to low-risk sport, so categories 2 and 3 were condensed to 1 low-risk category. The effect of adding tibial slope and deleting 55 cases from the full model is shown in Table 4.

**Table 4:** Odds Ratios (SE) for the Graft Rupture Model Before and After Including Tibial Slope, Adjusted for RTS Time and Level^*a*^

	Excluding Tibial Slope (n = 557)		Including Tibial Slope (n = 507)	
Predictor Variable	Odds Ratio	SE	Odds Ratio	SE
ACL + LET	0.31	0.12	0.34	0.15
Age	0.83	0.06	0.85	0.07
Female sex	0.97	0.31	1.30	0.46
Knee hyperextension	0.80	0.34	0.93	0.45
Graft diameter	0.80	0.24	0.75	0.26
Medial meniscal				
Repair	0.97	0.36	1.11	0.45
Excision	1.74	0.74	2.03	0.97
Lateral meniscal				
Repair	0.95	0.47	0.78	0.44
Excision	1.12	0.46	0.94	0.45
High-grade knee laxity	3.12	1.19	3.56	1.54
Exposure time	1.22	0.04	1.11	0.05
RTS level				
None (reference level)	—	—	—	—
Low risk, low level	1.41	1.66	1.12	1.42
High risk, low level	1.73	1.81	1.58	1.61
High risk, high level	1.81	2.17	1.58	1.99
Tibial slope	—	—	1.15	0.08

a ACL, anterior cruciate ligament; LET, lateral extra-articular tenodesis; RTS, return to sport. Dashes indicate no odds ratio available.

All assumptions were checked, identifying 10 potential outliers; however, removing these observations did not change the model estimates or affect statistical significance. The pared-down model with ORs and 95% CIs for important predictors of graft rupture is presented in Table 5.

**Table 5:** Predictors of Graft Rupture After Paring Down the Model Using *P <* .30^*a*^

Predictor Variable	Odds Ratio	95% CI	*P* Value^*b*^
ACL + LET	0.40	0.18-0.91	**.03**
Age	0.83	0.72-0.96	**.01**
Tibial slope	1.15	1.01-1.32	**.049**
High-grade knee laxity	3.27	1.45-7.41	**.004**
Medial meniscal excision	1.88	0.64-5.50	.25
Exposure time	1.18	1.08-1.29	**.001**

a Effect of age per 1-year increase. Effect of tibial slope per 1° increase. Effect of exposure time per 1-month increase. ACL + LET, high-grade knee laxity, and medial meniscal excision included as dichotomous variables (yes/no). ACL, anterior cruciate ligament; LET, lateral extra-articular tenodesis.

b Bold indicates statistical significance.

The LET procedure was significantly associated with 60% lower odds (95% CI, 0.18-0.91) of graft rupture, and a 1-year increase in age was associated with 17% lower odds of rupture (95% CI, 0.72-0.96). A 1° increase in posterior tibial slope was significantly associated with 15% higher odds (95% CI, 1.01-1.32) of rupture. Patients with high-grade preoperative knee laxity were at 3.27-times higher odds (95% CI, 1.45-7.41) of graft rupture. Exposure time remained in the model as a significant predictor, with each additional month of exposure time (ie, indicating an earlier RTS) increasing the odds of rupture by 18% (95% CI, 1.08-1.29). The Hosmer-Lemeshow test returned a nonsignificant result (*P* = .91), and the AUC for the ROC curve was 0.78.

**Figure 1.**
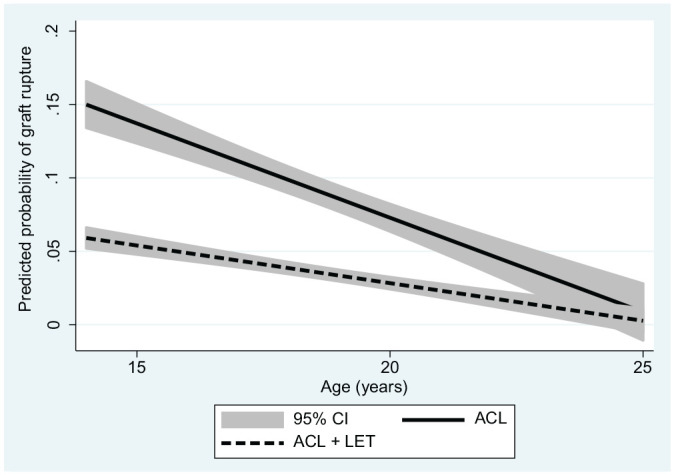
Predicted probability of graft rupture by age with and without the addition of a LET for patients in the Stability 1 Study, adjusted for tibial slope angle, medial meniscal deficiency, high-grade knee laxity, and time of return to sport. ACL, anterior cruciate ligament; LET, lateral extra-articular tenodesis.

**Figure 2.**
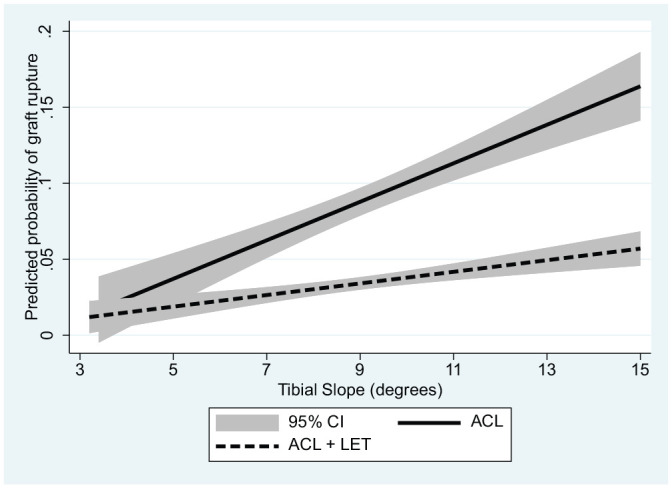
Predicted probability of graft rupture by tibial slope angle with and without the addition of a LET for patients in the Stability 1 Study, adjusted for age, medial meniscal deficiency, high-grade knee laxity, and time of return to sport. ACL, anterior cruciate ligament; LET, lateral extra-articular tenodesis.

**Figure 3.**
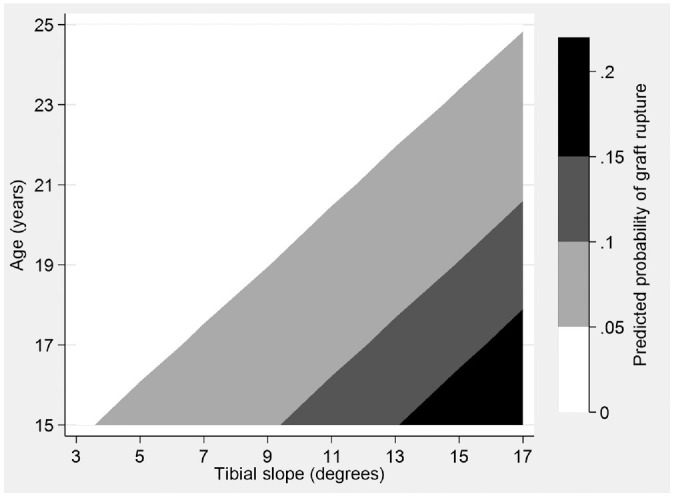
Contour plot showing predicted probabilities of graft failure for patients in the Stability 1 Study by age and tibial slope, adjusted for the addition of a LET, high-grade knee laxity, time returned to sport, and deficient medial meniscus. This shows the predicted probability of ACLR rupture from the Stability 1 Study as a function of tibial slope angle and patient age. Predicted probabilities range from approximately 0% to 25%, with greater probability of failure indicated by areas with darker shading. ACLR, anterior cruciate ligament reconstruction; LET, lateral extra-articular tenodesis.

Predicted probabilities of graft rupture for the 2 continuous predictors, age and posterior tibial slope, are presented graphically by group (Figures 1 and 2) as well as overall (Figure 3).

Table 6 summarizes important predictors that contributed to the final models (*P* < .30) and thus may be indications for a LET based on the goal of the procedure in young high-risk patients.

**Table 6:** Preoperative and Operative Indications for Adding a LET^*a*^

Variable	Asymmetric Pivot	Rupture
Younger age	×	×^*b*^
Knee hyperextension	×	
Small graft diameter	×^*b*^	
Medial meniscal		
Repair	×	
Excision	×	×
Greater tibial slope	×	×^*b*^
High-grade knee laxity		×^*b*^
Earlier return to sport		×^*b*^

a Preoperative and operative indications that adding a LET to hamstring autograft may be warranted to reduce the odds of asymmetric pivot shift or graft rupture. LET, lateral extra-articular tenodesis.

b Statistically significant.

Last, we performed a sensitivity analysis using an ROC curve to determine the optimal threshold of the posterior tibial slope variable that best identified those at greater risk of graft failure. The optimal threshold for tibial slope was 9.4° (AUC, 0.62), and patients with a slope >9.4° were at 2.7-times greater odds (95% CI, 1.28-5.76) of graft rupture than patients with a slope below this threshold.

## Discussion

This multivariable analysis has identified preoperative patient characteristics and surgical variables that are associated with persistent rotatory laxity and graft rupture within the Stability 1 Study randomized clinical trial. The most important finding was that augmentation of a hamstring tendon autograft ACLR with a LET reduces the odds of graft rupture by 60% and postoperative asymmetric pivot shift by 46% after adjusting for other confounding factors.

Age was tightly constrained within our cohort, as only skeletally mature patients who were ≤25 years old at the time of surgery were included. Regardless, younger age was associated with higher odds of asymmetric pivot shift and significantly higher odds of graft rupture after adjusting for RTS time (exposure) and level. Previous studies have also shown that younger patients are at higher risk of graft failure.^23,59^ Webster et al^59^ observed that the incidence of ACL failure in patients aged <20 years was 30% at 2 years when ipsilateral and contralateral injuries were combined. In a study from the MOON group, Kaeding et al^23^ demonstrated that young age was associated with ACL reinjury, with a 9% decrease in risk for every year gained in age. In a more recent study from the MOON group, college athletes aged <23 years had a 19.7% incidence of ACL rupture of either knee by postoperative 6 years.^53^ Furthermore, patients treated with a hamstring autograft had 2.1-times higher odds of rupture than those treated with a bone–patellar tendon–bone autograft.

In the Stability 1 Study, posterior tibial slope was a significant predictor of graft rupture and contributed to the model for asymmetric pivot shift. The mean tibial slope in our study was 9° (SD, 2.7°), which is similar to the mean slope from the case-control study by Webb et al^58^ (male, 9.3° [SD, 2.4°]; female, 8.5° [SD, 2.3°]), which found an association between slope and reinjury. The same group studied the association of tibial slope and age, demonstrating the “catastrophic” effect of young age and increased posterior tibial slope, with an 11-times increase in risk of graft rupture at postoperative 2 years if <18 years old with a tibial slope >12°.^45^ Our data showed that patients with a tibial slope above approximately 9.5° had over twice the odds of graft rupture as compared with patients with a slope below that threshold, although the risk difference between patients receiving ACLR alone and ACLR + LET was similar at all levels of tibial slope (Appendix Table A1, available in the online version of this article). If the effectiveness of a LET does not change with tibial slope (ie, LET is equally protective at all slope angles), this suggests that a LET may contribute to a different mechanism than tibial slope. While tibial slope is modifiable through osteotomy procedures, surgeons may first want to consider using less aggressive procedures known to reduce risk of graft rupture, such as a LET,^14,52^ or a different graft choice, such as bone–patellar tendon–bone autograft,^53^ even if these factors work independently.

To understand the significantly increased risk of ACLR failure related to age and tibial slope, we investigated the effectiveness of adding a LET visually. We plotted the predicted probability of graft rupture with 95% CIs across the range of tibial slope values and patients’ ages within the Stability 1 Study, adjusting for other important predictors. The graphs suggest that adding a LET to a hamstring tendon autograft ACLR significantly reduces the probability of failure in patients aged <23 years and with a tibial slope >6°, as the 95% CIs no longer overlap at these proposed thresholds. Tables showing graft rupture between groups (Appendix Tables A1 and A2, available online) demonstrate a similar relative risk at all values of slope and age, with the addition of a LET being 3 to 4 times more protective than a hamstring autograft ACLR alone.

In this study, lateral meniscal repair or excision had no effect on either outcome and therefore was excluded from the model. While medial meniscal tears requiring treatment at the time of surgery were not statistically significant in either model, they did remain as potential contributing variables: medial meniscal repair was retained in the model for asymmetric pivot shift, while medial meniscal excision was retained for asymmetric pivot shift and graft rupture.

In the asymmetric pivot-shift model, the odds of residual laxity were slightly greater when meniscal excision (OR, 1.55; 95% CI, 0.79-3.06) was performed as compared with meniscal repair (OR, 1.30; 95% CI, 0.85-1.99) in relation to no treatment of the medial meniscus. The menisci are understood to behave as secondary stabilizers to anterior translation and anterolateral subluxation in the ACL-deficient knee.^36^ Jacquet et al^20^ recently found a statistically significant association between meniscal treatment and high-grade residual laxity at postoperative 3.5 years. While they assessed meniscal treatment as a whole rather than the medial and lateral compartments separately, they demonstrated that the odds of residual laxity were 3.3 times greater in patients undergoing meniscal repair versus no repair and 2.7 times greater in patients undergoing meniscectomy versus repair.

Medial meniscal excision, while nonsignificant, was associated with 1.9-times higher odds (95% CI, 0.64-5.50) of graft rupture in our model. Despite suggestions that repair of a medial meniscal tear may lead to poor outcomes,^60^ this evidence supports the need for meniscal preservation during ACLR, as a deficient medial meniscus may be more problematic. Robb et al^42^ prospectively followed 124 patients undergoing primary ACLR over the course of 2 years and performed a survival analysis to determine prognostic factors of graft survival. Eighteen patients (14.5%) in that study experienced graft failure. In addition, medial and lateral meniscal deficiency was associated with the risk of failure, and the risk of failure was >4 times higher for those with medial meniscal deficiency. Research from the MOON group on concomitant meniscal tears at the time of ACL surgery revealed that while medial and lateral repairs fail at a similar rate by postoperative 6 years, medial retears occur earlier than lateral retears (mean, 2.1 vs 3.7 years).^60^ The SANTI Group found that in 383 patients followed for 2 to 5 years, the addition of an anterolateral ligament reconstruction at the time of ACLR was protective of medial meniscal repair, as the failure rate was 2 times lower in the group undergoing ACLR and anterolateral ligament reconstruction than ACLR alone.^51^ As such, anterolateral procedures such as LET or anterolateral ligament reconstruction may be protective of not only ACL graft rupture but also meniscal repair failure, which in turn may have a combined effect on ACLR outcomes. In contrast, there are concerns that the addition of an anterolateral procedure may overconstrain the knee joint, potentially leading to an increased risk of osteoarthritis (OA) development in the long term.^41^ In a 2017 systematic review of 8 studies and 421 patients, Devitt et al^9^ showed a low incidence of OA in the first 11 postoperative years after combined ACLR and LET; however, 2 studies with >24-month follow-up demonstrated OA rates >50%. Further evidence of long-term outcomes is required; thus, surgeons should weigh the potential risks and benefits when deciding whether to augment ACLR with a LET.

Unsurprisingly, increased exposure time (ie, earlier RTS) was associated with graft rupture. Multiple studies have demonstrated the negative effects of an early RTS.^5,16,19,38^ This may be secondary to reduced neuromuscular conditioning^39^ as well as the lack of ACL graft maturity.^57^ In a recent narrative review, Nagelli and Hewett^37^ posed the question of whether RTS should be delayed until at least 2 years postoperatively to allow for appropriate healing and rehabilitation. While this may not be a plausible option for many young athletes, it does highlight the need for better RTS assessment and functional testing before release of patients back to full activity. Understanding these factors warrants a conversation with patients regarding the addition of a LET but also to determine their postoperative goals, particularly surrounding their desire and intended timing of return to high-risk sports. Despite the lack of retention in the model, our analysis showed that return to a higher-risk sport carried greater risk than return to a lower-risk sport (see Table 4).

Graft diameter has been shown to be predictive of failed hamstring ACLR. Snaebjörnsson et al^47^ demonstrated that a graft size <8 mm was associated with failure, while a follow-up study by Spragg et al^54^ reported that a 0.5-mm increase in graft diameter was associated with a 14%-18% reduction in the likelihood of revision surgery. Graft diameter was not related to graft rupture in our study, likely in part because of the specific efforts made to control for graft size intraoperatively by tripling the semitendinosus tendon if a 4-strand semitendinosus/gracilis construct was <8 mm. However, even with these measures in place, we did find that a 1.0-mm increase in graft diameter was associated with 38% lower odds of asymmetric pivot shift.

Knee hyperextension contributed to the model for asymmetric pivot shift but was not predictive of graft rupture. Because hyperextension is linked with mechanisms that can increase graft laxity, such as impingement,^22^ superficial laceration,^44^ and increased tension on the ACL,^21^ it is not surprising that hyperextension is predictive of rotational instability but not rupture. Several biomechanical and cadaveric studies have assessed the effect of knee hyperextension on impingement and graft tension. Jagodzinksi et al^22^ performed magnetic resonance imaging scans of 15 knees and found strong correlation (*r* = 0.67; *P* = .006) between the degree of hyperextension and graft impingement. Goss et al^15^ took 5 fresh-frozen cadaveric knees and assessed contact pressure and graft tension for 3 tibial tunnel positions: they found (1) higher contact pressures between the ACL graft and the intercondylar notch as hyperextension increased and (2) increasing graft tension as the degree of hyperextension was greater, regardless of tunnel placement. Clinical research studies have shown conflicting results. Several studies have shown an association between knee hyperextension and preoperative instability,^4,43^ postoperative instability,^56^ and graft failure,^8^ while others report no relationship between hyperextension and risk of laxity or rupture.^7^

High-grade preoperative knee laxity, as determined by a grade 3 Lachman or pivot-shift test result, was significantly associated with 3-times greater odds of graft rupture within the Stability 1 Study, although it was not retained in the asymmetric pivot-shift model. The Stability 1 cohort was intentionally based on high-risk patients with significant preoperative laxity,^12^ including 62% of patients with grade 2 Lachman test and 76% of patients with grade 2 pivot-shift test. Thus, the low- vs high-grade variable is largely a comparison of grade 2 vs 3 laxity. Our findings suggest that patients with high-grade laxity have similar odds of residual rotational laxity as those with low-grade laxity, while high-grade laxity does contribute to the risk of graft rupture. In contrast, Jacquet et al^20^ recently showed that high-grade preoperative laxity was predictive of residual laxity in a cohort of 266 patients, although they were older (age, 18-50 years) and predominantly male (71.3%). Magnussen et al^29,30^ showed an association between high-grade laxity and graft rupture in the MOON cohort at postoperative 2 and 6 years. They also showed that high-grade laxity was not associated with the risk of contralateral ACL tear, suggesting that high-grade laxity was related to injury-specific rather than patient-specific factors.^29^

This study has limitations, particularly in relation to the patient sample included in the analysis. First, the sample was part of a randomized controlled trial of young active patients at high risk of retear who underwent ACLR with a hamstring autograft, exhibiting specific criteria that put them at higher risk of failure. As such, this cohort is not representative of the overall ACLR population or those receiving other graft types for ACLR. It is also not clear whether the addition of a LET to a bone–patellar tendon–bone autograft would provide the same level of protection, as found in the previously mentioned MOON study,^23^ and this issue is under investigation in our ongoing Stability 2 Study (ClinicalTrials.gov: NCT03935750). Second, while the pivot-shift test was scored by experienced members of the surgical team (ie, orthopaedic surgeons or surgical fellows), this was a multicenter study; thus, various members of the surgical team performed the pivot-shift assessment. While the assessment was performed according to the IKDC scoring system and the trial methodology required multiple grade 1 pivot-shift test results for the primary outcome to reduce the effect of measurement variability, there may be some differences in how the pivot shift was graded. Third, surgical variables such as tunnel placement have been identified as predictors of ACLR failure in previous studies but were not controlled for in the Stability 1 trial. Furthermore, given the relatively low number of graft ruptures (n = 45), we are at risk of overfitting the rupture model, as logistic regression requires at least 10 events per predictor to be adequately powered.^40^ Validation outside the study sample is particularly important for a model with few events; however, the predictors identified by this analysis do coincide with clinical hypotheses for graft rupture.^11,50^

## Conclusion

The addition of a LET to hamstring autograft ACLR was significantly associated with 60% lower odds of graft rupture, while younger age, increased tibial slope, high-grade preoperative knee laxity, and early RTS were associated with higher odds of graft rupture. Younger age, knee hyperextension, increased tibial slope, and medial meniscal repair or excision were all related to higher odds of asymmetric pivot shift, while adding a LET and increasing hamstring autograft diameter significantly reduced asymmetric pivot shift in our high-risk cohort from the Stability 1 Study. Orthopaedic surgeons should consider supplementing hamstring autograft ACLR with a LET in young active patients with morphological characteristics that make them at high risk of reinjury, as the LET was protective when adjusted for other variables in this analysis.

## Authors

Andrew D. Firth, MSc, Dianne M. Bryant, MSc, PhD, Alan M.J. Getgood, MD, Robert Litchfield, MD (London Health Sciences Centre, Western University, Fowler Kennedy Sport Medicine Clinic, London, Canada); Robert G. McCormack, MD (Fraser Orthopaedic Institute, New Westminster, Canada); Mark Heard, MD (Banff Sport Medicine, Banff, Canada); Peter B. MacDonald, MD (Pan Am Clinic, Winnipeg, Canada); Tim Spalding (University Hospitals Coventry Warwickshire NHS Trust, Coventry, UK); Peter C.M. Verdonk, MD, PhD (Antwerp Orthopaedic Center, Ghent, Belgium); Devin Peterson, MD (McMaster University, Hamilton, Canada); Davide Bardana, MD (Queens University, Kingston, Canada); Alex Rezansoff, MD (Sport Medicine Centre, University of Calgary, Calgary, Canada); and the STABILITY Study Group (Kevin Willits, MD, Trevor Birmingham, PhD, Chris Hewison, MD, Stacey Wanlin, Ryan Pinto, MSc, Ashley Martindale, MSc, Lindsey O’Neill, MSc, Morgan Jennings, MSc, Michal Daniluk, MSc, [London Health Sciences Centre, Western University, Fowler Kennedy Sport Medicine Clinic, London, Canada]; Dory Boyer, MD, Mauri Zomar, CCRP, Karyn Moon, Raely Moon, Brenda Fan, Bindu Mohan [Fraser Orthopaedic Institute, New Westminster, Canada]; Gregory M. Buchko, MD, Laurie A. Hiemstra, MD, PhD, Sarah Kerslake, MSc, Jeremy Tynedal, MPH [Banff Sport Medicine, Banff, Canada]; Greg Stranges, MD, Sheila Mcrae, PhD, LeeAnne Gullett, Holly Brown, BHK, Alexandra Legary, Alison Longo, Mat Christian, Celeste Ferguson [Pan Am Clinic, Winnipeg, Canada]; Nick Mohtadi, MD, Rhamona Barber, Denise Chan, MSc, Caitlin Campbell, Alexandra Garven, BSc, Karen Pulsifer, Michelle Mayer [Sport Medicine Centre, University of Calgary, Calgary, Canada]; Nicole Simunovic, MSc, Andrew Duong, MSc, David Robinson, David Levy, Matt Skelly, BSc, Ajaykumar Shanmugaraj, BSc [McMaster University, Hamilton, Canada]; Fiona Howells, BPharm, Murray Tough [Queens University, Kingston, Canada]; Pete Thompson, Andrew Metcalfe, Laura Asplin, Alisen Dube, Louise Clarkson, Jaclyn Brown, Alison Bolsover, Carolyn Bradshaw, Larissa Belgrove, Francis Milan, Sylvia Turner, Sarah Verdugo, Janet Lowe, Debra Dunne, Kerri McGowan, Charlie-Marie Suddens [University Hospitals Coventry Warwickshire NHS Trust, Coventry, UK]; Geert Declerq, MD, Kristien Vuylsteke, and Mieke Van Haver [Antwerp Orthopaedic Center, Ghent, Belgium]).

## Supplemental Material

sj-pdf-1-ajs-10.1177_03635465211061150 – Supplemental material for Predictors of Graft Failure in Young Active Patients Undergoing Hamstring Autograft Anterior Cruciate Ligament Reconstruction With or Without a Lateral Extra-articular Tenodesis: The Stability Experience Click here for additional data file. Supplemental material, sj-pdf-1-ajs-10.1177_03635465211061150 for Predictors of Graft Failure in Young Active Patients Undergoing Hamstring Autograft Anterior Cruciate Ligament Reconstruction With or Without a Lateral Extra-articular Tenodesis: The Stability Experience by Andrew D. Firth, Dianne M. Bryant, Robert Litchfield, Robert G. McCormack, Mark Heard, Peter B. MacDonald, Tim Spalding, Peter C.M. Verdonk, Devin Peterson, Davide Bardana, Alex Rezansoff, STABILITY Study Group, Alan M.J. Getgood, Kevin Willits, Trevor Birmingham, Chris Hewison, Stacey Wanlin, Ryan Pinto, Ashley Martindale, Lindsey O’Neill, Morgan Jennings, Michal Daniluk, Dory Boyer, Mauri Zomar, Karyn Moon, Raely Moon, Brenda Fan, Bindu Mohan, Gregory M. Buchko, Laurie A. Hiemstra, Sarah Kerslake, Jeremy Tynedal, Greg Stranges, Sheila Mcrae, LeeAnne Gullett, Holly Brown, Alexandra Legary, Alison Longo, Mat Christian, Celeste Ferguson, Nick Mohtadi, Rhamona Barber, Denise Chan, Caitlin Campbell, Alexandra Garven, Karen Pulsifer, Michelle Mayer, Nicole Simunovic, Andrew Duong, David Robinson, David Levy, Matt Skelly, Ajaykumar Shanmugaraj, Fiona Howells, Murray Tough, Pete Thompson, Andrew Metcalfe, Laura Asplin, Alisen Dube, Louise Clarkson, Jaclyn Brown, Alison Bolsover, Carolyn Bradshaw, Larissa Belgrove, Francis Milan, Sylvia Turner, Sarah Verdugo, Janet Lowe, Debra Dunne, Kerri McGowan, Charlie-Marie Suddens, Geert Declerq, Kristien Vuylsteke and Mieke Van Haver in The American Journal of Sports Medicine
