# Supplementary material for: Predictors of Graft Failure in Young Active Patients Undergoing Hamstring Autograft Anterior Cruciate Ligament Reconstruction With or Without a Lateral Extra-articular Tenodesis: The Stability Experience
Source: Am J Sports Med. 2022 Jan 20;50(2):384–95. doi: 10.1177/03635465211061150 (PMC8829733; doi:10.1177/03635465211061150)
Supplement: sj-pdf-1-ajs-10.1177_03635465211061150 – Supplemental material for Predictors of Graft Failure in Young Active Patients Undergoing Hamstring Autograft Anterior Cruciate Ligament Reconstruction With or Without a Lateral Extra-articular Tenodesis: The Stability Experience [file sj-pdf-1-ajs-10.1177_03635465211061150.pdf]

Predictors of Graft Failure in Young, Active Patients Undergoing Hamstring Autograft Anterior Cruciate Ligament Reconstruction with or without an LET: The Stability Experience

Appendix – Additional Tables

Appendix Table A1 - Relative risk of graft rupture by group below or at and above each threshold of tibial slope in patients from the Stability I Study

Slope	Cat	ACL Intact	ACL Rupture	LET Intact	LET Rupture	Risk Ratio
**5**	<5	17	2	19	0	-
	5+	245	31	261	11	0.36
**6**	<6	35	3	41	0	-
	6+	227	30	239	11	0.38
**7**	<7	62	5	55	1	0.24
	7+	200	28	225	10	0.35
**8**	<8	87	5	84	1	0.22
	8+	175	28	196	10	0.35
**9**	<9	118	8	133	2	0.23
	9+	144	25	147	9	0.39
**10**	<10	154	11	171	4	0.35
	10+	108	22	109	7	0.36
**11**	<11	177	15	200	6	0.38
	11+	85	18	80	5	0.34
**12**	<12	202	17	227	7	0.39
	12+	60	16	53	4	0.33
**13**	<13	220	21	241	7	0.32
	13+	42	12	39	4	0.42
**14**	<14	228	22	250	7	0.31
	14+	34	11	30	4	0.48
**15**	<15	236	23	252	8	0.35
	15+	26	10	28	3	0.35

Appendix Table A2 – Relative risk of graft rupture by group below or at and above each threshold of patient age in patients from the Stability I Study

Age	Cat	ACL Intact	ACL Rupture	LET Intact	LET Rupture	Risk Ratio
**15**	<15	7	1	11	0	-
	15+	254	32	267	11	**0.35**
**16**	<16	31	7	40	3	**0.38**
	16+	238	8	250	26	**0.32**
**17**	<17	79	16	75	4	**0.30**
	17+	182	17	203	7	**0.39**
**18**	<18	113	23	114	7	**0.34**
	18+	148	10	164	4	**0.38**
**19**	<19	138	25	143	9	**0.39**
	19+	123	8	135	2	**0.24**
**20**	<20	158	27	160	10	**0.40**
	20+	103	6	118	1	**0.15**
**21**	<21	181	30	184	10	**0.36**
	21+	80	3	94	1	**0.29**
**22**	<22	201	31	198	10	**0.36**
	22+	60	2	80	1	**0.38**
**23**	<23	215	32	223	10	**0.33**
	23+	46	1	55	1	**0.84**
**24**	<24	225	32	241	11	**0.35**
	24+	35	1	37	0	**-**
